# Supplementary figures and images for: Analysis of transcripts and splice isoforms in red clover (Trifolium pratense L.) by single-molecule long-read sequencing
Source: BMC Plant Biol. 2018 Nov 26;18:300. doi: 10.1186/s12870-018-1534-8 (PMC6258457; doi:10.1186/s12870-018-1534-8)

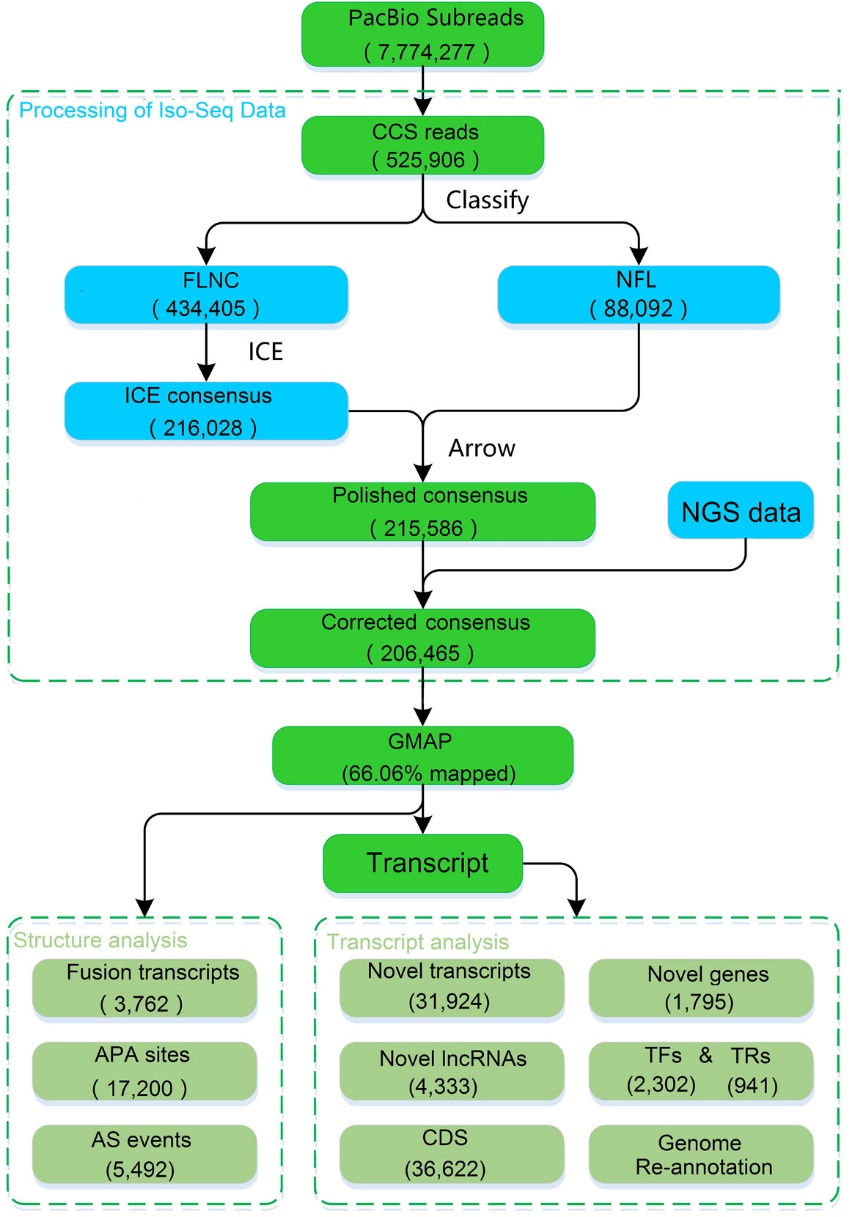


**Figure S1 Flow chart of Bioinformation analysis.**

Supplement: Supplementary file 1 — Figure S1. Flow chart of bioinformation analysis. (DOCX 241 kb) [file 12870_2018_1534_MOESM1_ESM.docx]
