# Supplementary material for: Analysis of transcripts and splice isoforms in red clover (Trifolium pratense L.) by single-molecule long-read sequencing
Source: BMC Plant Biol. 2018 Nov 26;18:300. doi: 10.1186/s12870-018-1534-8 (PMC6258457; doi:10.1186/s12870-018-1534-8)
Supplement: Supplementary file 6 — Figure S2. RT-PCR validation of AS and isoforms in 5 candidate genes. Arrows, PCR products; M, DNA Marker DL2000 Plus II; 1, Novelgene0860; 2, Tp57577_TGAC_v2_gene10390; 3, Tp57577_TGAC_v2_gene11337; 4, Tp57577_TGAC_v2_gene11508; 5, Novelgene0380. (DOCX 228 kb) [file 12870_2018_1534_MOESM6_ESM.docx]

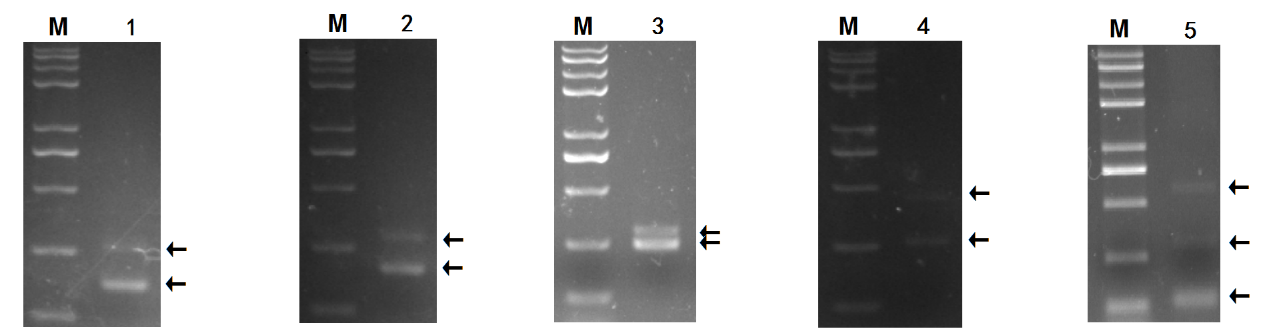


**Figure S2. RT-PCR validation of AS events and isoforms in 5 candidate genes.** Arrows, PCR products; M, DNA Marker DL2000 Plus II; 1, Novelgene0860; 2, Tp57577_TGAC_v2_gene10390; 3, Tp57577_TGAC_v2_gene11337; 4, Tp57577_TGAC_v2_gene11508; 5, Novelgene0380.
