# Supplementary material for: Analysis of transcripts and splice isoforms in red clover (Trifolium pratense L.) by single-molecule long-read sequencing
Source: BMC Plant Biol. 2018 Nov 26;18:300. doi: 10.1186/s12870-018-1534-8 (PMC6258457; doi:10.1186/s12870-018-1534-8)
Supplement: Supplementary file 9 — Figure S3. RT-PCR validation of 10 fusion transcripts. M, DNA Marker; 1, i0_HQ_c62675/f4p0/1006; 2, i3_LQ_c25970/f1p0/3394; 3, i2_HQ_c22922/f2p46/2488; 4, i1_LQ_c86101/f1p0/1505; 5, i2_LQ_c40661/f1p0/2186; 6, i2_LQ_c60404/f1p0/2058; 7, i0_LQ_c129257/f1p0/896; 8, i0_LQ_c8517/f3p0/608; 9, i1_HQ_c82285/f12p0/1057; 10, i1_LQ_c24613/f1p2/1285. (DOCX 687 kb) [file 12870_2018_1534_MOESM9_ESM.docx]

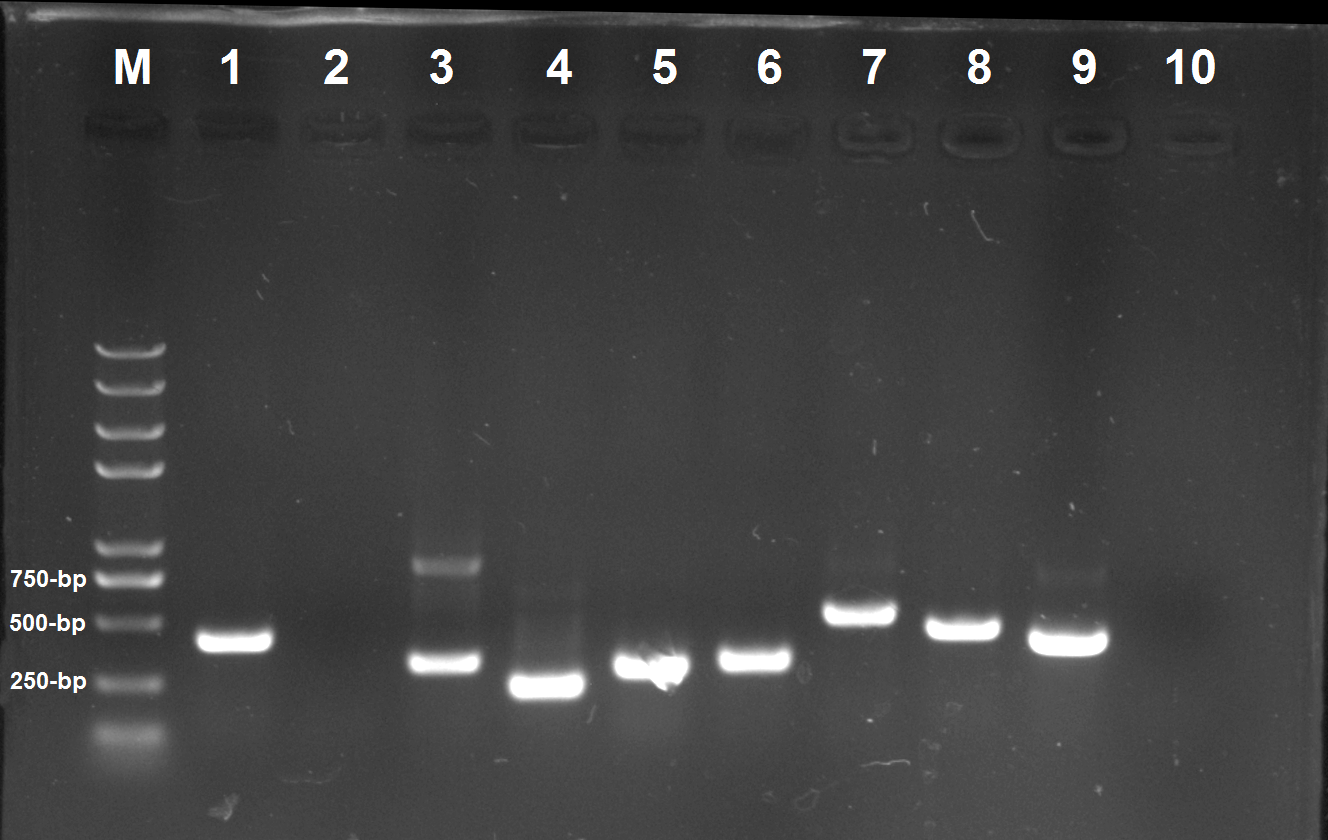


**Figure S3. RT-PCR validation of 10 fusion transcripts.** M, DNA Marker DL2000 Plus II; 1, i0_HQ_c62675/f4p0/1006; 2, i3_LQ_c25970/f1p0/3394; 3, i2_HQ_c22922/f2p46/2488; 4, i1_LQ_c86101/f1p0/1505; 5, i2_LQ_c40661/f1p0/2186; 6, i2_LQ_c60404/f1p0/2058; 7, i0_LQ_c129257/f1p0/896; 8, i0_LQ_c8517/f3p0/608; 9, i1_HQ_c82285/f12p0/1057; 10, i1_LQ_c24613/f1p2/1285
